# Relevance of the semi-quantitative short Food Frequency Questionnaire in assessment of calcium consumption by female inhabitants of Zabrze over the age of 55 years (the Silesia Osteo Active Study)

**DOI:** 10.1007/s11657-019-0620-3

**Published:** 2019-06-29

**Authors:** Katarzyna Martela, Roman Kuźniewicz, Wojciech Pluskiewicz, Elżbieta Tabor, Piotr Zagórski

**Affiliations:** 1 Doctoral Studies, School of Medicine with the Division of Dentistry, Medical University of Silesia in Katowice, Plac Traugutta 2, 41-800 Zabrze, Poland; 2 Department and Clinic of Internal Diseases, Diabetology, and Nephrology, School of Medicine with the Division of Dentistry, Metabolic Bone Diseases Unit, Medical University of Silesia in Katowice, 1 Maja 13-15, 41-800 Zabrze, Poland; 3 Department and Clinic of Internal Diseases, Diabetology, and Nephrology, School of Medicine with the Division of Dentistry, Medical University of Silesia in Katowice, 1 Maja 13-15, 41-800 Zabrze, Poland; 4 Department of Orthopaedic Surgery, Sports-Clinic, Bankowa 2, 44-240 Zory, Poland

**Keywords:** Food Frequency Questionnaire, Calcium, Women, Poland, Osteoporosis, Food record

## Abstract

***Summary*:**

In the study, we compare two methods used to assess the effects of nourishment on the bone status. Statistical analysis demonstrated a moderate conformity of both methods. Short Food Frequency Questionnaires can be used as short medical screening tool for calcium intake among women over 55 years of life.

**Introduction:**

Osteoporosis is a civilisation disease, the development of which is, among others, controlled and affected by diet. The factors which promote the health of bones include calcium, vitamin D, vitamin K, phosphorus, magnesium, and protein. A number of nutritional epidemiology methods can be applied to assess the effects of nourishment on the bone status, e.g. Food Frequency Questionnaire (FFQ) in its full or short (sFFQ) version or 3-day food record (3DFR). Both methods are known and widely used tools.

**Methods:**

In the reported study, we attempted to compare and assess the sFFQ and 3DFR tools. Both methods were employed to examine 156 women, the majority of whom presented with an overweight-indicating BMI. An analysis of sFFQ data brought an observation that most of the studied patients (33.3%) consumed milk once a day.

**Results:**

Based on 3DFR and sFFQ, we compared the measured volumes of consumed calcium which were 557.8 mg/day and 880.7 mg/day, respectively. The Cohen’s kappa calculator was used for a diagnostic evaluation of both tools; the kappa index was 0.5047, demonstrating a moderate conformity of both methods. In addition, sensitivity and specificity indices were calculated, revealing the values of 97% and 12%, respectively.

**Conclusions:**

sFFQ can be used as a short medical screening. It is suggested to use both the 3DFR, conducted by the patient, and sFFQ, as a complementary method. It is necessary to continue this type of studies and to standardise the methods of nutritional status assessment with regard to selected groups.

## Introduction

Osteoporosis is a disease that is characterized by low bone mass, deterioration of bone tissue, and disruption of bone microarchitecture: it can lead to compromised bone strength and an increase in the risk of fractures [1]. Osteoporosis is one of the civilisation diseases, the onset and course of which depend on the diet with an appropriate supplementation of nutritional components, i.e. calcium, phosphorus, magnesium and vitamin D3 or protein [2–5].

Among the methods of its prevention and slowing down, the course of this illness is physical activity, healthy lifestyle and proper diet.

Calcium is an essential element in the human body and is necessary to various cell functions. Calcium is not only important to bone health, but it is also essential for neuromuscular activity, blood coagulation and normal cardiac function. It is a vital component of skeleton where it is deposited by osteoblasts on a bone matrix throughout life. Food-derived calcium is absorbed in small intestine to blood plasma where its level is controlled by parathyroid glands. In case of low calcium level, parathyroids stimulate increased resorption of calcium in the kidneys and intestines, accelerating by that bone resorption. Therefore, an adequate intake of calcium is necessary to maintain this balance and healthy bones. [6, 7]

To assess the way of nutrition, it is necessary to use reliable, repeatable and simple diagnostic tools.

Researchers are making attempts to evaluate the relationships which seem to occur between diet components and the development of given disease entities. Reference methods are usually employed to assess the kind of diet, such as the method of keeping records from one or more days, a 24-h history method, analytical methods or biomarkers [8]. Furthermore, the validated Food Frequency Questionnaire begins to be more and more often used in nutritional epidemiological studies [9].

The aim of this reported study was a comparison of the 3-day food record method (3DFR) with the short, semi-quantitative Food Frequency Questionnaire (sFFQ). Then, the suitability for use of either method was analysed in medical outpatient environment.

A definition of a valuable, short method for patients’ diet assessment may allow to design preliminary, nutritional, medical screening programmes, complemented by properly selected prophylactic measures and patient’s reference to a clinical dietician.

## Methods

### Material

A nutrition analysis was carried out at the Department of Metabolic Diseases of the Medical University of Silesia in Zabrze in April and May 2015. A research project launch was preceded by a consent, provided local ethic committee. The number of female inhabitants of Zabrze over the age of 55 years was appr. 30,000. In order to obtain a representative population, a staff of Municipal Authority of Zabrze used systematic sampling by which they selected 10% of whole group. Therefore, a population including 3000 women was ready for further steps of project realisation [10]. Such kind of recruitment allows to state that our population was a statistically representative sample of local female population. Therefore, a method of subject selection allows to obtain an epidemiological, representative female sample.

Self-administered invitations were sent by mail to every woman in aforementioned group, and 388 responses were received. For purpose to conduct the study, each woman after personal registration attended the confirmation appointment.

**Fig. 1:**
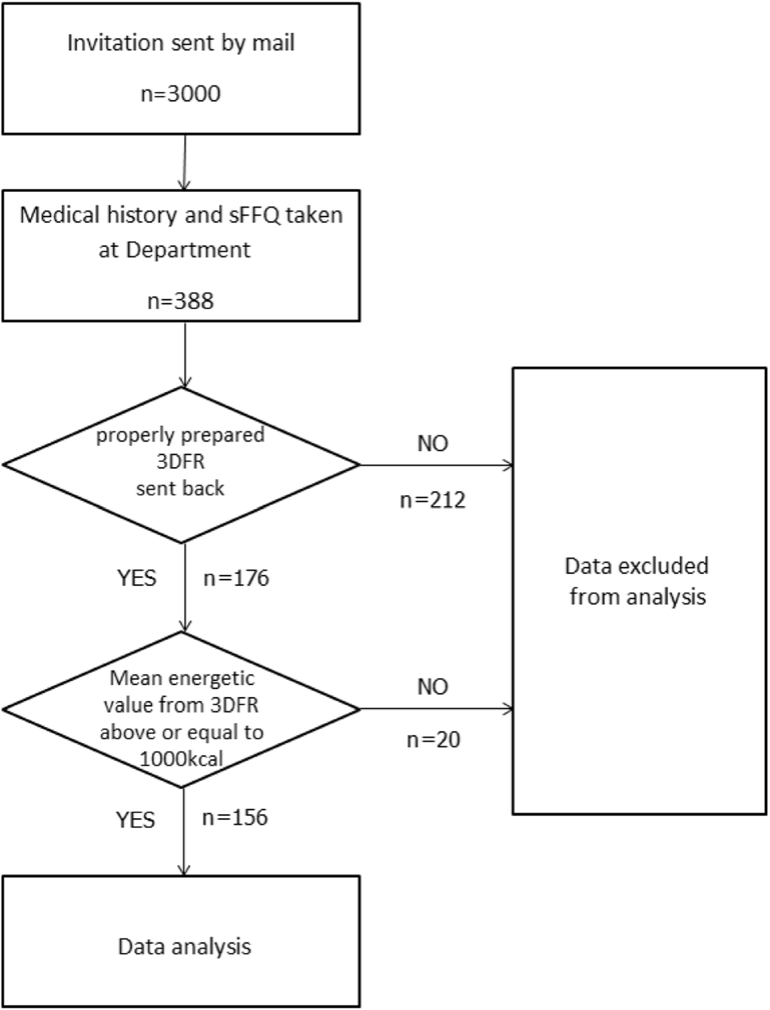
Population selection flowchart

Prior to the final enrolment, all the women provided their conscious consent to participate in the study. A short medical history was obtained from all the women by authors, followed by a nutrition part. The dietetic part consisted of a sFFQ and a 3-day food record (3DFR). It should be emphasised that all the patients were subject of sFFQ, while 176 patients submitted their 3-day food records. The data of 156 women (40.2% of the group) were transferred to further processing, since the energetic value from food records of 20 studied women was below 1000 kcal, what we considered to be a kind of a systematic error in the study apparatus [11, 12]. Figure 1 shows population selecting method.

The performed evaluation of nutrition habits employed sFFQ and 3DFR tools, where sFFQ consisted of 12 closed questions (see Table 2). The questions were selected with regard to the products with positive effects on the bone system (rich in vitamin-mineral components important for bones). The benchmark for the questionnaire was FFQ-6 (Food Frequency Questionnaire-6), designed by Prof. L. Wądołowska which is a validated tool used to assess the diet of the Polish population [13]. The answers to all the questions in the sFFQ questionnaire covered 6 food frequency categories, i.e.

- Never/almost never
- Once a month or more rarely
- Several times a month
- Several times a week
- Every day
- Several times a day

Used sFFQ is a validated tool for assessing calcium in Polish population [9]. The sFFQ questionnaire was completed with questions concerning the portion size of dairy products. Thus, it could be used not only for qualitative evaluation but also for the semi-quantitative estimation. The numerical values of calcium supplementation were obtained by multiplying the number and size of portions by the proportion coefficient, resulting from consumption frequency. Regarding the products, other than dairy products, the size of consumed portion was defined as the portion size which was most frequently recorded in 3DFR and which corresponded to a given item in sFFQ [8]. Calcium contents in the products were obtained from the table of nutritional values of selected products and typical meals issued by Polish Food and Nutrition Institute (Instytut Żywności i Żywienia—IŻŻ) [14].

The patients after performing sFFQ by authors were then asked to complete the 3DFR diary. Records were to be collected from two working days (not occurring one after the other) and from one weekend day. In order to obtain all the necessary data, each of the patients was provided with a diary instruction. The received diaries were entered into an Aliant dietetic calculator (Anmarsoft, Poland). The nutritional values of the products ad meals, used by the computer program, came from a revised database of the Institute of Food and Nutrition. The contents of nutritional components in the products were obtained from the same table of nutritional values of selected products and typical meals as in sFFQ.

### Data analyses

The obtained data were reviewed with regard to their completeness and appropriateness and then entered into a database, configured in the MS Excel 2010 program (Microsoft, USA) and submitted to statistical analysis by means of the Statistica 12.0 software package (StatSoft, USA) [15]. Descriptive statistics was initially calculated. The normality of distribution was verified, using the Shapiro-Wilk’s test. The descriptive statistics, presented in Tables 1 and 3, demonstrate the mean values and standard deviations (SD) for the data which demonstrated normal distribution. In order to compare the conformity of the two methods, i.e. the 3DFR diary and the sFFQ questionnaire, their correlation was checked by means of the Pearson and Spearmen correlation coefficients and by the Bland-Altman plot. According to purpose of dietary evaluation as discrimination if calcium intake is adequate, the group was divided into the following two classes to obtain a diagnostic evaluation of the test: the patients, consuming below 1000 mg of calcium per day, and the patients consuming ≥ 1000 mg/day—the value was selected on the basis of the Estimated Age Requirement (EAR) for calcium. Then, Cohen’s kappa statistics was used to verify tests grouping repeatability.

**Table 1:** Characteristic features of the study group (*n* = 156)

Factor	Mean value ± SD	Median value	Range
Age (years)	65*	64	55–87
Body mass (kg)	74.3 ± 12.7	72.5	43.5–108
Body height (cm)	158.1 ± 5.6	158	143.5–172.5
BMI (kg/m^2^)	29.8 ± 5.1	28.7	17.3–42.2

*BMI* body mass index

*Age distribution is not normal distribution

Sensitivity and specificity were calculated using Newcomb formula, basing on cross-classification according whether a test indicated the presence of calcium daily intake above EAR page. All values of *p* < 0.05 were regarded as statistically significant [16–18].

## Results

### Group characteristics

Table 1 presents general characteristics of the group (156 women). The mean age for women in that group was 65 years, while the mean body mass and height values were 74.3 kg and 158.1 cm, respectively. The mean BMI (body mass index) was 29.8.

### Calcium supplementation values

The data, collected by means of sFFQ, are presented in Table 2. Analysing the frequency of consumption of milk and of dairy products, we may easily note that the majority of the questioned patients reported milk consumption frequency of once daily, while the frequency of consumption of dairy products was even lower.

**Table 2:** sFFQ—a short questionnaire of consumption frequency of dairy products and of other selected food products (summery of provided answers, *n* = 156)

	Never	1–3 Times a month	Once a week	A few times a week	Once daily	A few times during the day
How often do you drink milk?	46 (29.5%)	9 (5.8%)	5 (3.2%)	30 (19.2%)	52 (33.3%)	14 (9%)
How often do you consume fermented dairy products (e.g. kefir, yoghurt)?	9 (5.8%)	19 (12.2%)	19 (12.2%)	58 (37.2%)	47 (30.1%)	4 (2.6%)
How often do you consume hard, ripened and processed cheeses?	19 (12.2%)	17 (10.9%)	19 (12.2%)	78 (50%)	16 (10.3%)	7 (4.5%)
How often do you consume marine fish (e.g. salmon, mackerel, and sardines)?	7 (4.5%)	43 (27.6%)	81 (51.9%)	25 (16%)	0	0
How often do you consume preserved meat and fish products?	97 (62.2%)	33 (21.1%)	23 (14.7%)	3 (1.9%)	0	0
How often do you consume any of the specified products: parsley, red pepper or sprouts?	3 (1.9%)	17 (10.9%)	24 (15.4%)	68 (43.6%)	39 (25%)	5 (3.2%)
How often do you consume soy or soy-based products (e.g. soy pâté, chops or tofu)?	135 (86.5%)	15 (9.6%)	2 (1.3%)	4 (2.6%)	0	0
How often do you eat sausages or smoked meat?	4 (2.6%)	9 (5.8%)	21 (13.5%)	66 (42.3%)	48 (30.8%)	8 (5.1%)
How often do you consume whole grain products (including, among others, oat flakes, rye flour bread on leavening, whole grain bread)?	20 (12.8%)	13 (8.3%)	9 (5.8%)	39 (25%)	43 (27.6%)	32 (20.5%)
How often do you consume lettuce or other green leafy vegetables and green vegetables?	6 (3.8%)	12 (7.7%)	20 (12.8%)	67 (42.9%)	44 (28.2%)	7 (4.5%)
How often do you consume fruit?	3 (1.9%)	4 (2.6%)	4 (2.6%)	26 (16.7%)	72 (46.2%)	47 (30.1%)
How often do you consume seeds (sunflower, pumpkin) or nuts (almonds, walnuts)?	48 (30.8%)	34 (21.8%)	16 (10.3%)	33 (21.1%)	23 (14.7%)	2 (1.3%)

*sFFQ -* short Food Frequency Questionnaire

The mean daily calcium supplementation values, calculated by the two methods, differed between each other when compared using a paired Student’s *T* test. Namely, it was 557.8 mg in 3DFR records and 880.7 mg in sFFQ data (see Table 3). These values are statistically different at *p* < 0.05.

**Table 3:** Calcium supplementation volumes, assessed by 3DFR and sFFQ (*n* = 156)

	Mean value	Median value	Range
Mean daily calcium supplementation* (mg/day)	557.8 ± 228.8	527.9	185.6–1291
Calcium volume in sFFQ (mg/day)	880.7 ± 578.5	735.5	41.3–3653.5

*The mean daily supplementation means the arithmetic mean (of a given nutrition component), calculated for 3 days (based on 3DFR). *p* < 0.05

*sFFQ* short Food Frequency Questionnaire

Calcium consumption SD was 228.8 mg for 3DFR and 578.5 mg for sFFQ (see Table 3).

### sFFQ and 3DFR compliance in calcium intake assessment

#### Correlation and linear regression

**Fig. 2:**
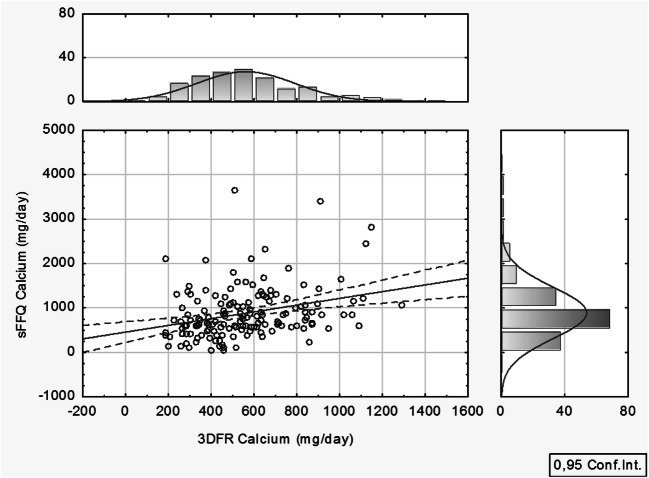
Graphical presentation of Pearson’s correlation with the level of confidence at 0.95 (*n* = 156)

A statistical (comparative) analysis of either method demonstrated a correlation between the calcium volumes, calculated from 3DFR, and the calcium quantities, obtained from a semi-quantitative sFFQ, what was confirmed by the positive Pearson’s correlation coefficient (*r* = 0.30) at the level of significance of *p* < 0.05 (see Fig. 2).

A linear regression analysis enabled to assess the standard error of 3DFR-assayed calcium value estimation by the semi-quantitative sFFQ analysis. This value may be approximated by the following formula:

$$ \left[3\mathrm{DFR}\ \mathrm{Ca}\right]=453.24+0.12\times \left[\mathrm{sFFQ}\ \mathrm{Ca}\right] $$

#### Bland Altman plot

**Fig. 3:**
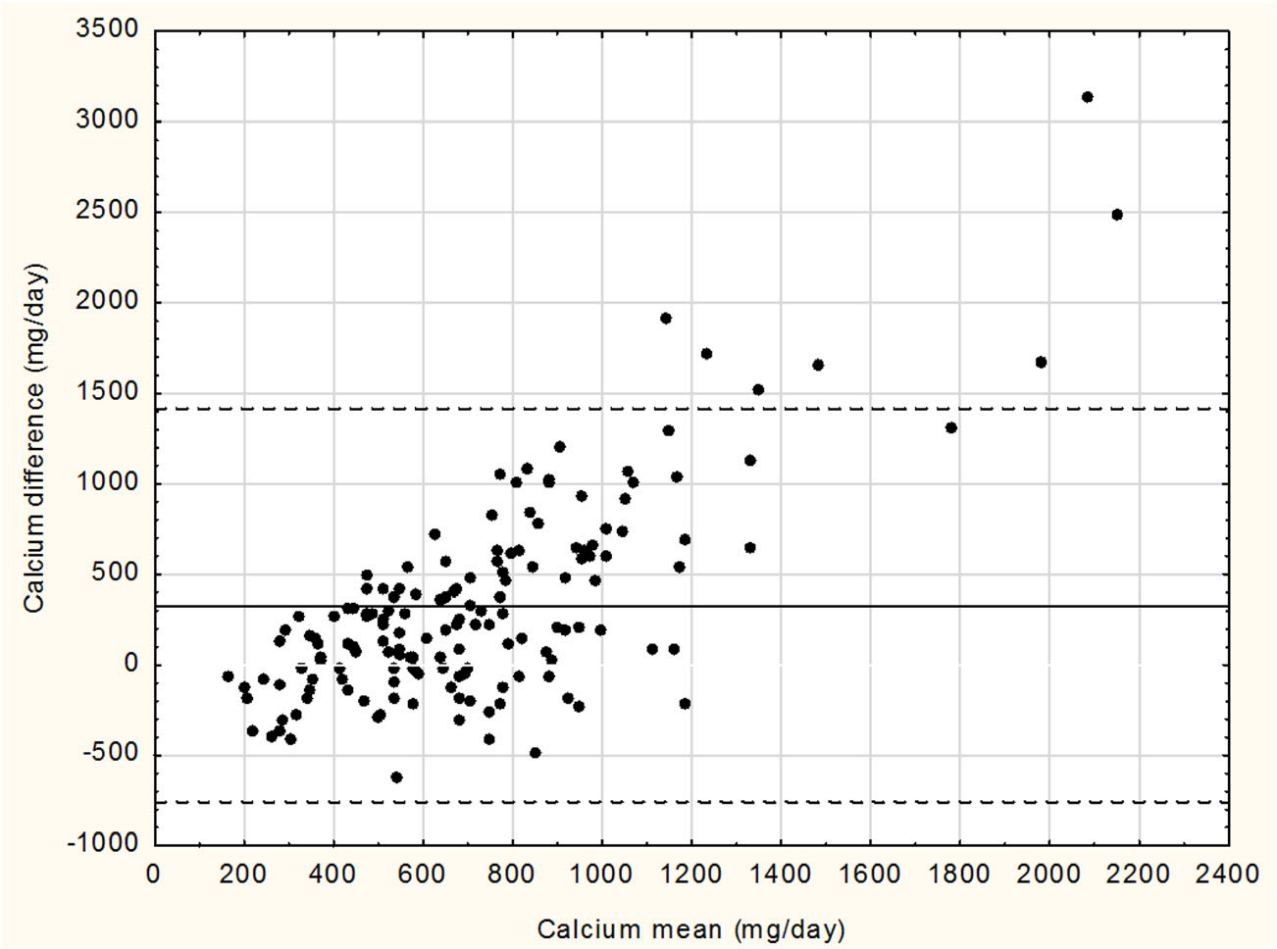
Bland-Altman plot of the difference between the methods (sFFQ and 3DFR) against their mean (*n* = 156). The mean (solid line) and the ± 2 SD (broken lines) of the differences are shown

The Bland-Altman plot, comparing the calcium volumes, calculated from 3DFR, with sFFQ-assessed quantities, indicated the value below 5% (see Fig. 3). It means that both methods indicate full compliance, since at least 95% of the differences between either measurement are within the interval of ± 2 SDs from the mean difference.

#### Cohen’s kappa

Using the Cohen’s kappa statistics calculator, the kappa coefficient was obtained for our study at the level of 0.5047. According to available scales used in kappa statistics interpretation, the obtained result indicates a moderate and acceptable conformity of both methods.

In addition, the sensitivity and specificity indices were calculated, revealing the values of 97% and 12%, respectively. While assessing the diagnostic value of the tests, the sensitivity index was higher from the specificity index, which implies that the methods better identify persons with calcium deficits in their diet.

## Discussion

A proper nutrition scheme, perceived as a necessary prophylactic element, is of particular importance for patients with the risk of osteoporosis [19, 20]. A multitude of analyses, both at national and international level, demonstrates too little calcium consumption in various populations [21, 22]. A very good example confirming this thesis is given in a study by Australian researchers, indicating a correlation between the consumption of calcium in food stuffs and a decreased bone fracture risk [23, 24]. Our research group also revealed too little calcium supplementation in relation to the standards recommended by the Polish Institute of Food and Nutrition in 2017. The Institute of Food and Nutrition recommends the Estimated Average Requirement (EAR) to be 1000 mg/day for that group of women [25].

Therefore, it is very important to find a reliable method, enabling a quick assessment of the consumption levels of all the components which are important for healthy bones. An analysis, carried out by Magarey et al., was a review of 36 assessment tools, which are applied to evaluate the consumption levels of calcium and dairy products (including the Food Frequency Questionnaire—FFQ—for dairy products, FFQ for calcium-rich products, an on-line FFQ for 15 and 25 products or a shortened FFQ). The analysis demonstrated that the used methods were not reliable and should thus be used with due care. The kappa statistics characterised some of the methods at the level of 0.8, while the value was lower in case of our study. Nevertheless, the authors of the mentioned report suggest that when selecting the method of evaluation, it makes sense to take into account the type, size, age, sex and physiological condition of a population. It may then be worthwhile to classify nutrition evaluation methods in the context of various studied groups [26]. A Croatian study of 333 postmenopausal women has demonstrated that FFQ may not be suitable for determination of either low, i.e. < 500 mg or very high, i.e. > 1200 mg, calcium supplementation. Similarly, as in the study of Dickson et al., we may observe from the Bland-Altman plot that FFQ is not suitable for individual calcium consumption assessment for its rather broad range of distribution. The cited study used data from a 27-component sFFQ and from a 24-h history. A comparison of the methods gave the kappa coefficient of 0.43 [27].

An analysis, performed by Angel M. Ong et al. in a group of 108 postmenopausal women, showed FFQ as a simple tool for calcium consumption assessment within 600–1000 mg, as well as for calcium assays in epidemiological studies [28]. A study similar to our internal research was conducted by Jensen in 162 Asians, Spaniards and white population subjects, all of them being the inhabitants of the USA. That group was requested to fill an FFQ questionnaire, based on 80 products, to reveal the amount of consumed calcium. In addition, FFQ was completed in the 2nd and the 3rd week with a 24-h history. The dependence of calcium supplementation, determined by FFQ and obtained from a twice-repeated 24-h history, attained 0.54 in Pearson’s coefficient (*r*). It demonstrates a strong correlation of the results, obtained by means of the two methods [29]. While making a review of the collected studies, one may perceive a high similarity, while there is no specific research management algorithm which would allow for unification of the research procedure, followed by a more precise evaluation. The nutrition evaluation methods, used by other researchers in the above-mentioned publications, differ from our apparatus with the following: the selection of method type, accounted food products in FFQ, the number of questions, the repetition or not of the 24-h food record, or the method of questionnaire and food record concepts (together with patient or on-line).

All the mentioned factors could possibly have had some impact on the final results and conclusions. An example of analysis with a methodology concept, different from those in the above presented studies, is a study of a group of women in the Northern Africa. The researchers made an attempt to estimate the volumes of consumed vegetables and fruit (as an element of balanced diet and a kind of prophylactics against non-infectious diseases). In this regard, a three times repeated 24-h nutrition record and a short FFQ (8-components), also repeated twice, were employed (which was not the case for other authors). As the outcome of the analysis demonstrated, that specific questionnaire of food consumption frequency turned out to be a reliable tool in the assessment of consumed vegetables and fruit by a population of 100 women, inhabiting the areas of the Northern Africa [30]. Another research project, this time of Australian researchers was based on a comparison of a 9-part FFQ (marked as MFQ—containing products rich in omega-3, plus a 74-part semi-quantitative FFQ). Both questionnaires were validated. As it was demonstrated by obtained results, MFQ may be a quick tool to identify consumed products, rich in omega-3. It is not recommended in terms of an individual analysis but may be an extremely useful tool at population level [31].

FFQ is a tool which can qualitatively estimate nutritional habits, and, when it is completed with questions, concerning the quantity and size of food portions, it becomes useful to determine (semi-quantitatively) the supplementation of vitamins or mineral components.

A nutrition diary is a cheap and simple method for evaluation (the record method). Such a record book usually covers, at least, 3 days. It provides information on nutrition methods and, by conducted actual recording, specifies the consumed quantities of nutrients. This method may, however, introduce a number of errors into the study. The character of errors depends mainly on the accuracy of the surveyed respondent. Among others, a tendency is often observed to record what is, in the respondent’s understanding, optimal in a given study (thus, the notes can be underestimated and inadequate vs. the actual, consumed portions, e.g. by disregarding unhealthy snacks). In addition, keeping food consumption records demands the patient’s writing and reading skills, enforces his/her ability to assess consumed portions and is, eventually, labour-intensive and tiresome [32, 33].

It should be emphasised that the results of nutrition analyses may, in effect, be more or less reliable, which, in turn, indicates the necessity of proper evaluation method selection, depending on a given situation. A validation process of the selected method, which determines its repeatability and reliability, is also an important element.

From the point of view of nutrition, a dietician has in his/her practice a possibility (tools) to run a full nutritional analysis and diagnosis; he/she may also repeat them during follow-up visits. However, it is a long-term and experience-demanding process, taking into account the fact that the usually limited time at a doctor’s office does not allow for a full nutrition analysis.

## Conclusions

1. The sFFQ questionnaire can be used for evaluation of probable calcium deficits in diet—as a short medical screening tool.
2. Both FFQ and the 3-day food record (3-DFR) can be regarded as tools appropriate for nutrition evaluation of various population groups.
3. Taking into account the above analysis, as well as the analyses of publications by other researchers, it cannot be unambiguously determined whether the small Food Frequency Questionnaire (sFFQ) may replace the 3-day food record.
4. It is thus suggested that both the food record, maintained by the patient, and FFQ or sFFQ are conducted, the latter being approached as a complementary method.
5. There is a need of further studies and of the selection and standardisation of nutrition status assessment methods, with an indication of groups where they are applicable.

## Abbreviations

EAR: Estimated age requirement
FFQ: Food Frequency Questionnaire
3DFR: 3-Day food record
sFFQ: Short Food Frequency Questionnaire
SUM: Silesian University of Medicine
BMI: Body mass index
FFQ-6: Food Frequency Questionnaire-6
SD: Standard deviations
r: Pearson’s coefficient
MFQ: Marine omega-3 FFQ
